# Gastrokine 1 mRNA in human sera is not informative biomarker for gastric cancer

**DOI:** 10.1186/s12952-016-0057-9

**Published:** 2016-07-25

**Authors:** Valentina Villano, Chiara Stella Di Stadio, Antonella Federico, Filomena Altieri, Giuseppina Miselli, Maurizio De Palma, Emilia Rippa, Paolo Arcari

**Affiliations:** Department of Molecular Medicine and Medical Biotechnology, University of Naples Federico II, Via S. Pansini 5, I-8031 Naples, Italy; Hospital A. Cardarelli, Nasples, Italy; CEINGE, Advanced Biotechnology scarl, Naples, Italy

**Keywords:** Absolute qRT-PCR, 18 kDa antrum mucosal protein, Gastric cancer biomarker, Gastrokine 1

## Abstract

**Background:**

We aimed to ascertain if Gastrokine 1 mRNA in the sera of patients with gastric cancer might be an informative biomarker for the disease.

**Results:**

Analysis of GKN1 mRNA in serum samples from healthy individuals (*n* = 23) and from patients with diagnosis of gastric cancer (*n* = 16), performed by using absolute quantification based on standard curve method, did not show any significative statistical difference between the two unpaired group of individuals.

**Conclusions:**

Our preliminary results did not confirm GKN1 as a potential biomarker for gastric cancer.

## Background

Gastric cancer (GC) is still one of the prevalent leading causes of cancer-related deaths worldwide and high mortality rate is mainly due to late-stage diagnosis [2]. Survival rate at 5 years of about 20 % in most areas of the world [6, 11, 32], except in Japan where mass screening programs, staging systems, and treatment may contribute to superior 5-year survival rates of approximately 60 % [30, 35, 37]. In all Europe, the incidence is about 104,620 and 69,394 among males and females, respectively [16], representing about 23 % of all cancers.

The most common type of stomach cancer is adenocarcinoma, which is divided into intestinal (well-differentiated) and diffuse (undifferentiated) each having different epidemiological and pathophysiological features [20]. The intestinal-type generally evolves through a relatively well-defined sequence of histological lesions, namely non-atrophic gastritis, chronic atrophic gastritis, intestinal metaplasia, and dysplasia [4, 5]. On the contrary, the diffuse-type has instead a poorer prognosis and develops through unknown genetic and morphological events from normal gastric epithelium. The pathogenesis of GC remains poorly understood however several environmental factors, such as *Helicobacter pylori* (*H. pylori*) infection can be the cause leading to this disease. This risk is probably the result of a combination of genetic and environmental factors in which the infection by *H. pylori* is of particular relevance.

In a previous work, we analyzed the protein profile of malignant and normal gastric tissues and identified a novel stomach specific protein gastrokine 1 (GKN1) whose expression was reduced in *H. pylori* infected gastric mucosa and down-regulated or completely absent in GC tissues and precancerous lesions. [13, 28]. GKN1 belongs to a family of genes encoding stomach-specific proteins formed by 3 known members: GKN1 [23], GKN2 [9], and GKN3 [26]. These proteins, besides a highly conserved structure, show convergent functions in terms of modulation of gastric mucosal homeostasis and inflammation, activity in epithelial wound healing and/or repair, and anti-proliferative activity. Moreover, they are highly expressed in the normal stomach and loss of GKNs expression in gastric cancers suggests putative tumor suppressor roles [24]. For instance, *GKN2* knockout mice showed defective gastric epithelial differentiation whereas, loss of GKN2 in gp130^F/F^ caused tumorigenesis of the proximal stomach. Furthermore, in H. pylori–infected GKN2 knockout mice, gastric immunopathology was accelerated and associated with augmented T helper cell type 1 (Th1) [25]. However, GKNs modes of action remain unsolved. Some findings indicated the involvement of GKN1 in the replenishment of the surface lumen epithelial cell layer, and in maintaining mucosal integrity [36]. After injury of the gastric mucosa, restoration may occur very rapidly in the presence of GKN1. In contrast, if the protein is down-regulated the repair process may be hampered [19]. Application of GKN1 to gastrointestinal cells promoted epithelial restoration and exerted its protective effect by increasing accumulation of specific tight and adherens junction proteins and also protecting their loss after injury [33].

Under this consideration, GKN1 might represent an important biomarker in carcinogenic process since it was seen that individuals with a lower expression of the protein have an increased risk to develop gastric diseases. We hypothesized that GKN1 mRNAs identified in serum of GC patients could become a completely non-invasive biomarker potentially distinguishing GC patients from healthy individuals.

## Results and discussion

GKN1, also called Antrum Mucosal Protein of 18 kDa (AMP-18) [40], is considered one of the most putative gastric cancer biomarkers [43]. Its low expression can be concluded in onset of malignancy. Moreover, while its presence in *H. pylori* negative patient is normal, it is decreased in *H. pylori*-positive patients [28]. Because human serum is a rich source of biochemical products that can act as indicators of the physiological or clinical status of a patient, we exploited the potentiality of GKN1 as gastric cancer biomarker trying to highlight its presence in human serum. We first used Western blotting technique to analyze human sera of healthy individuals. Because of the unusually high abundance of human serum albumin (HSA) in serum that can interfere with the resolution and sensitivity of several proteome techniques, samples were partially purified by Centricon 30 to remove high molecular weight proteins and then analyzed by Western blotting comparing the intensity of the signals with that of human gastric mucosal extract. Detection with monoclonal mouse anti-GKN1 antibody did not show any positive band signals compared to that shown by human gastric mucosal extract (from data not shown). This result was also confirmed by analyzing the same serum samples with ELISA kit for GKN1. Although its lower limit of detection (0.056 ng/mL), no absorbance was observed in all sera analyzed (from data not shown). Because the given detection limit of both GKN1 antibody and ELISA kit is about 0.15 ng/mL (using as control recombinant GST tagged GKN1), the lack of detection of GKN1 in human sera, unless it is totally absent, might probably depends by a far lower concentration of the protein. Therefore, more sensitive methods for the detection of the protein in the serum should be used in order to ascertain this possibility.

**Fig. 1:**
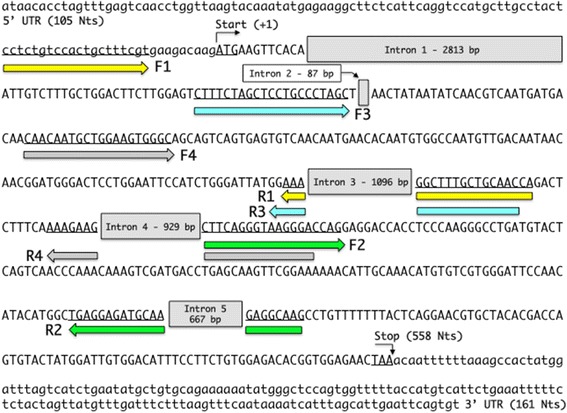
Design of PCR primers for the detection of GKN1 mRNA in human sera. Structure of *GKN1* gene and nucleotide sequence of GKN1 mRNA. The coding region is represented in capital letters, 5′ and 3′ UTR are in lower case, start and stop codons are underlined, intron regions are boxed. PCR primers pairs F1-R1, F2-R2, F3-R3 and F4-R4 are highlighted in yellow, green, azure and gray, respectively

**Fig. 2:**
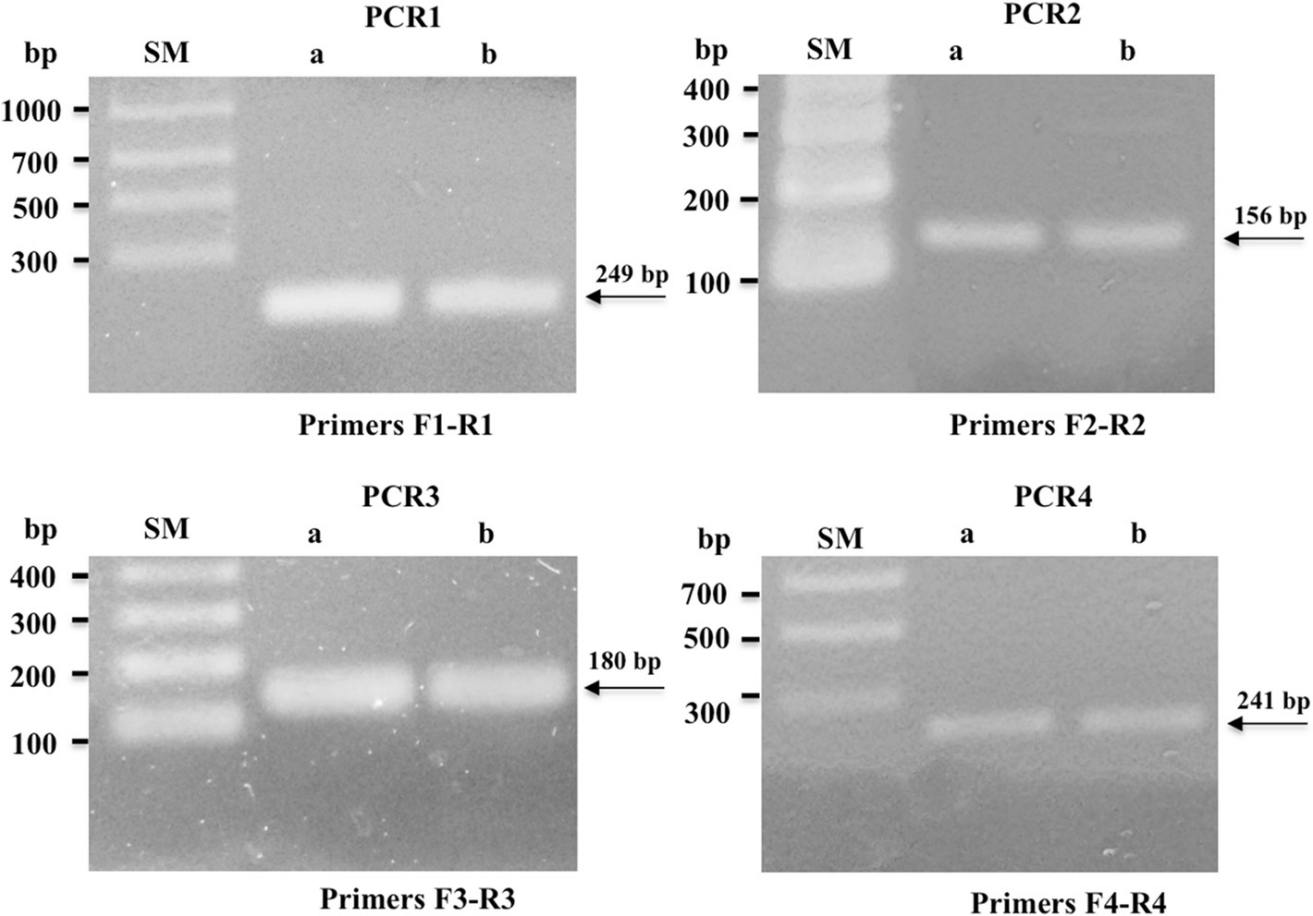
Semiquantitative PCR of GKN1 mRNA expression in human sera. Representative semiquantitative PCR performed on a serum of a healthy subject performed before (*lanes a*) and after (*lanes b*) storage of the sample at 4 °C for 24 h. PCR reactions (PCR1, PCR2, PCR3 and PCR4) were carried out using the primers pairs F1-R1, F2-R2, F3-R3 and F4-R4 as reported in Fig. 1

The above results prompted to search for GKN1 at transcription level. It is well known that mRNA in body fluid such as blood, has been proved as a novel resource to replace conventional tools for disease identification [15, 21, 27], and successfully used as cancer-related biomarker [1, 31, 39]. In fact, mRNA markers have been the targets for identifying patients with colorectal, breast, lung, and thyroid cancers, and malignant melanoma [3, 12, 17, 18, 40]. All these published studies were performed for testing a single mRNA marker, however no much is known about the nature of circulating extracellular RNA (exRNA), including mRNA, and the possible mechanisms by which these RNAs are protected from plasma RNase activity [10]. One possible hypothesis is that exRNA present in plasma/serum are stabilized through binding to protein/lipoprotein complexes or that that are sequestered within lipid vesicles [14]. In this scheme, we tried to assess if GKN1 circulating mRNA in serum might represent a biomarker for gastric cancer detection. Therefore, we first tried to detect the presence of GKN1 mRNA in sera of healthy volunteers. Four sets of PCR primers (F1-R1, F2-R2, F3-R3 and F4-R4), covering GKN1 coding sequence at exon and intron bounda [32] of *GKN1* gene, were defined by using PrimerBlast Server (Fig. 1) and used to perform semi-quantitative PCR on fresh human sera. As reported in Fig. 2 (lanes a), all primer couples were able to amplify the corresponding GKN1 cDNA region giving PCR products of the expected length. This finding suggested the presence of a full GKN1 mRNA in the samples analyzed and the absence of possible PCR products deriving from genomic DNA. Because RNA is widely thought to be labile in the circulation, as reported by [38], we then analyzed the same blood samples that had been left at 4 °C for 24 h. As shown in Fig. 2 (lanes b), no difference was observed in the band intensities after repeating the PCR in the same conditions, thus confirming the general stability of mRNAs [38]. To check if the four PCR products were corresponding to GKN1 cDNA, PCR sequencing analyses were performed. The results confirmed that the nucleotide sequences of the four PCR products were encoding GKN1 (not shown).

**Fig. 3:**
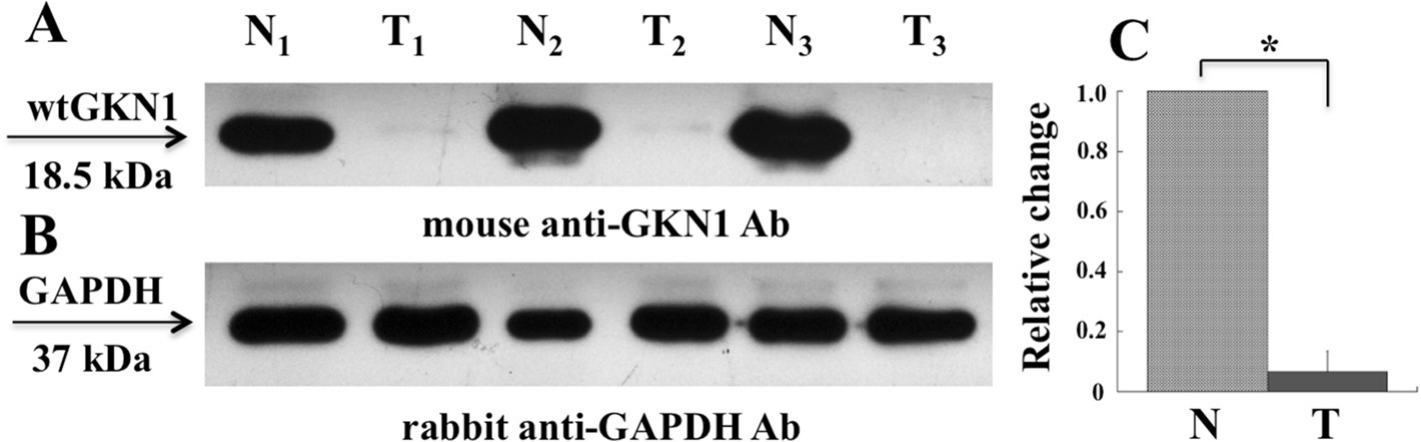
Expression levels of GKN1 in human gastric tissues. **a** Representative Western blot of equal amounts of cell extracts (20 μg) analyzed in paired non-tumoral (N_1_, N_2_, N_3_) and tumoral (T_1_, T_2_, T_3_) human gastric samples using mouse anti-GKN1 (**a**) and rabbit anti–GAPDH (**b**) antibodies (Ab). **c** qRT-PCR analysis. Total RNA was prepared from gastric tissues and analyzed by qRT-PCR for GKN1 mRNA level. Samples: N and T, non-tumoral and tumoral gastric tissues, respectively. Data from three experiments are reported as mean values ± SD. * *P* < 0.05

Therefore, we decided to use the quantitative real-time PCR (qRT-PCR) strategy to detect GKN1 mRNA in the serum obtained from a population of wealthy subject and in that from patients with diagnosis of gastric cancer. We took advantage from a collection of sera withdrawn from patients with a diagnosis of gastric cancer that underwent surgical treatment. The clinicopathologic characteristics of the gastric cancer patients enrolled in this study are outlined in Table 1. The intestinal type was well differentiated in one, moderately differentiated in three and poorly differentiated in the remaining cases, while as far as concerns stage, these were all advanced. Diffuse type GC was poorly differentiated and advanced in all cases. The non-tumoral areas of intestinal type GC showed a variable degree of atrophy with diffuse IM, instead, the non-tumoral areas of diffuse type GC showed a variable degree of non-dysplastic inflammation. Characterization of non-tumoral gastric mucosa (N) from tumoral (T) one was based on the macroscopic aspect of the normal tissue compared to the tumoral one, as evaluated from the hospital pathologist. Moreover, paired specimens of non-tumoral (N) and tumoral (T) gastric mucosa where analyzed by Western blotting for GKN1 expression levels. As shown in Fig. 3, compared to non-tumoral tissues (N), in the corresponding tumoral tissues (T) it was observed almost a complete down-regulation of GKN1 at protein level [8]. To assess whether the GKN1 down-regulation occurred also at mRNA level, qRT-PCR analyses were performed on total RNA extracted from three paired non-tumoral and tumoral gastric tissues (Fig. 3c). GKN1 mRNA is one of the most abundant transcripts known in the normal stomach (about 1 % of total gastric mRNA), often undetectable in gastric tumor tissues by Northern blotting [29]. However, qRT-PCR is a more sensitive method able to unveil the presence of low levels of a given mRNA in cells/tissues [7]. As shown in Fig. 3c, compared to non-tumoral tissues, qRT-PCR showed a decrease of GKN1 mRNA level in tumoral tissues [42]. Therefore, based on these findings, our hypothesis was that compared to healthy subjects, in sera of patients with GC, lower significative levels of GKN1 mRNA could have been evaluated by quantitative analysis.

**Table 1:** Characteristics of Healthy Subjects and Gastric Cancer Patients

Variable	Healthy^a^	Gastric Cancer
	Subjects (*n* = 23)	Subjects (*n* = 16)
Age (years)	59 ± 25	64 ± 13
Sex ratio (M:F) 8:23 1:6a	14/9	9/7
Tumor type	--	Intestinal 9, Diffuse 7
Grade of differentiation	--	Well 1, Moderate 3, Poor 12
Stage	--	Early 0 (0 %); Advanced 16 (100 %)

^a^All healthy individuals included did not have dyspeptic symptoms

**Fig. 4:**
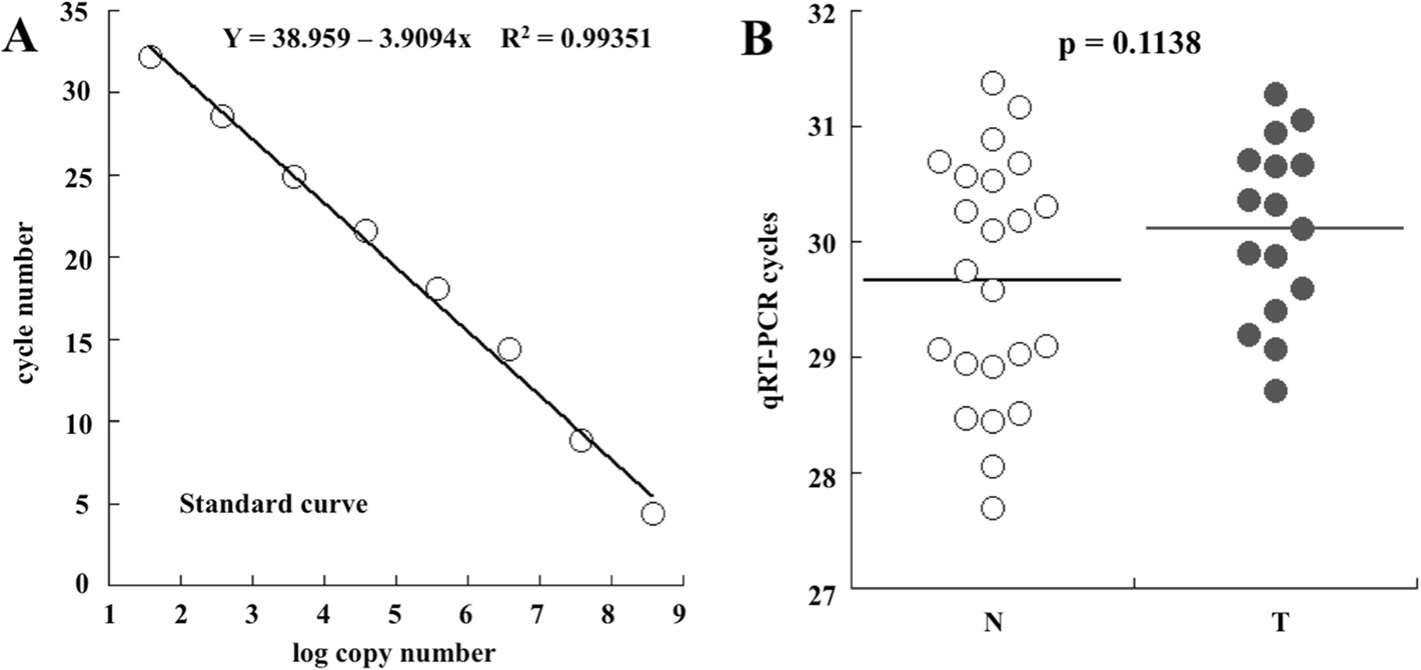
qRT-PCR of GKN1 mRNA levels in human sera. **a** Standard calibration curve constructed using pCDNA3.1 vector containing the cDNA encoding human GKN1 (pCDNA3.1-flGKN1). **b** Statistical evaluation of the results of the qRT-PCR performed on human sera from a population of healthy individuals (N) and from patients with gastric cancer (T)

qRT-PCR was performed by absolute quantification by a standard curve method constructed using as reference DNA a plasmid containing flGKN1 cDNA [34] and as primers the F2-R2 pair ( Fig. 4). We used serial 10-fold dilutions of plasmids to construct a standard curve by plotting the logarithm of the plasmid copy number against the measured cycle values. The standard curve had a wide range of DNA copies/μl (from 3.78 × 10^1^ up 3.78 × 10^8^) with a linear correlation (R^2^) of 0.99351, and a slope of −3.909 (Fig. 4a). The level of circulating GKN1 mRNA of all 23 healthy samples showed a median cycle number value of 29.6673 (range 27.702–31.380). GC patients gave a median Ct value of 30.1269 (range 28.713–31.279). The statistical evaluation of the results using Student *t*-Test of unpaired data with unequal variance indicated a non-significant difference between the two groups (*p* = 0.1138). From the standard curve, the mean circulating copies of GKN1 mRNA were 1.81x10^5^ and 2.38 × 10^5^ copies/ml in patients and healthy individuals, respectively (Fig. 4b). Despite the strong down-regulation of GKN1 protein levels in gastric cancer, the quite comparable levels of GKN1 mRNA strongly indicated that this down-regulation is not observed at transcription levels thus suggesting translation regulation mechanism for GKN1 expression. The result obtained suggested to direct the search towards other non-invasive gastric cancer biomarker such as long noncoding RNAs (lncRNAs), a recently discovered class of noncoding RNAs (ncRNA) that are emerging as a promising new class of biomarkers for tumour diagnosis [41].

## Conclusion

In conclusion, although we have shown for the first time the presence of GKN1 mRNA in human serum of healthy subjects as well as in that of patients with diagnosis of gastric cancer, when we analyzed by qRT-PCR these sera, we did not observe significant statistical differences among the two population. Therefore, we could not confirm our hypothesis that GKN1 mRNA could serve as non-invasive marker for GC.

## Methods

### Population

The study population comprised 16 patients with GC recruited at Hospital A. Cardarelli, Naples, Italy. All volunteers (23 individuals) were subjected to careful anamnesis and all those who had dyspeptic symptoms were excluded. Patients were interviewed regarding smoking habit, alcohol intake and chronic use of drugs and informed consent from each patient was obtained. Hospital Pathologist performed the macro dissection of tumor and non-tumor tissues of GC patients during surgery. Blood samples from these patients were collected before surgery and soon stored at −20 °C. Gastric cancer was classified according to Lauren criteria [20]. The study reported in the manuscript has been carried out in the frame of a research protocol entitled “Role of gastrokine 1 in gastric cancer” that has the approval from the Ethic Committee of the University of Naples Federico II (Comitato Etico Università Federico II). The assigned protocol number of the study was 34/15.

### Western blotting

Proteins from cell extracts were analyzed by Western blotting using mouse anti-GKN1 monoclonal antibody, clone 2E5 (Abnova, Taipei, Taiwan) at a dilution of 1:500. Detection was performed using the enhanced chemiluminescence detection kit (SuperSignal West Pico) following manufacturer’s instructions. All films were analyzed by using the Image J software. Western blot band intensity was measured with ImageJ 1.46r software.

### Serum samples for Western blotting and PCR analyses

Serum samples from healthy or gastric cancer subjects were prepared immediately after blood samples withdrawn and stored at −80 °C. For Western blotting, 1 mL of serum was diluted 1:1 with H_2_O and centrifuged at 4,000 rpm in Centricon 30 (Millipore, Darmstadt, Germany) to separate higher size proteins. Filtered sample was then lyophilized overnight, re-suspended in 50 μL H_2_O and analyzed by Western blotting using mouse anti-GKN1 antibody, or by GKN1 ELISA Kit (Cloud-Clone Corp, Huston, USA), according to the manufacturer instruction.

Total RNA from 1 mL of serum sample was prepared using miRNeasy Serum/Plasma Kit (Qiagen). RNA concentration was measured using the NanoDrop 1000 spectrophotometer (Thermo Fisher Scientific). 1 μg of total RNA was retro-transcribed with the iScript cDNA Synthesis Kit (Bio-Rad, Milan). 4 μl of cDNA were amplified with 1 unit of Taq DNA Polymerase (Invitrogen) in the buffer provided by the manufacturer not containing MgCl_2_. Reactions were carried out in the PTC-0150 Mini-Cycler (Biorad) according to the following condition: first cycle of 5 min at 95 °C followed by 30 cycles (30 s at 95 °C, 40 s at 58 °C, 30 s at 72 °C) and 10 min at 72 °C. GAPDH was used as a control.

For semi-quantitative PCR, the following primers were designed: F1, cctctgtccactgctttcgt, R1, tggttgcagcaaagccattt; F2, cttcagggtaagggaccag, R2, cttgcctcttgcatctcctca; F3, ctttctagctcctgccctagc, R3, gttgcagcaaagccatttcc; F4, caacaatgctggaagt gggc, R4, tcccttaccctgaagttcttt.

For qRT-PCR, GKN1 cDNA was amplified using as primers F2-R2. qRT-PCR was performed with the SYBR Green PCR MasterMix (Applied Biosystems) under the following conditions: 10 min at 95 °C, followed by 40 cycles (15 s at 95 °C and 1 min at 60 °C). Each reaction was performed in duplicate. We used the 2^–ΔΔCT^ method to calculate the relative expression levels [22]. Results on serum samples were evaluated by absolute quantitation using a standard curve constructed using dilution of plasmid pCDNA3.1 containing GKN1 cDNA (pCDNA3.1-flGKN1) [34].

qRT-PCR and Western blotting from gastric tissues. Total RNA was extracted from non-tumoral and tumoral human tissues using TRIzol reagent solution (Invitrogen) according to the manufacturer’s protocol. GKN1 cDNA was amplified by qRT-PCR using F3-R3 primers, as above reported. Protein extracts from gastric tissues were prepared and analyzed by Western blotting as described [8].

### Statistical analyses

Statistical analyses were performed by two-tailed unpaired or paired Student’s *t*-test using KaleidaGraph 4.1.1 software. Data were reported as means ± standard deviation (SD). The significance was accepted at the level of *p* < 0.05.

## Abbreviations

GAPDH, glyceraldehyde-3-phosphate dehydrogenase; GC, gastric cancer; GKN1, gastrokine 1; HAS, human serum albumin
